# Consequences of spinal pain: Do age and gender matter? A Danish cross-sectional population-based study of 34,902 individuals 20-71 years of age

**DOI:** 10.1186/1471-2474-12-39

**Published:** 2011-02-08

**Authors:** Charlotte Leboeuf-Yde, René Fejer, Jan Nielsen, Kirsten O Kyvik, Jan Hartvigsen

**Affiliations:** 1 The Research Department, the Spine Centre of Southern Denmark, Hospital Lillebaelt, Middelfart, Denmark; 2 Institute of Regional Health Services Research, Faculty of Health Sciences, University of Southern Denmark, Odense, Denmark; 3 Center for National Clinical Databases, South, Department of Research and Health Technology Assessment, Odense University Hospital, Denmark; 4 Odense Patient data Exploratory Network (OPEN), Odense University Hospital, Odense, Denmark; 5 Institute of Sport Science and Clinical Biomechanics, Faculty of Health Sciences, University of Southern Denmark, Odense, Denmark; 6 Nordic Institute of Chiropractic and Clinical Biomechanics, Odense, Denmark

## Abstract

**Background:**

While low back pain (LBP) and neck pain (NP) have been extensively studied, knowledge on mid back pain (MBP) is still lacking. Furthermore, pain from these three spinal areas is typically studied or reported separately and in depth understanding of pain from the entire spine and its consequences is still needed.

**Objectives:**

To describe self-reported consequences of pain in the three spinal regions in relation to age and gender.

**Methods:**

This was a cross-sectional postal survey, comprising 34,902 twin individuals, representative of the general Danish adult population. The variables of interest in relation to consequences of spinal pain were: Care-seeking, reduced physical activity, sick-leave, change in work situation, and disability pension.

**Results:**

Almost two-thirds of individuals with spinal pain did not report any consequence. Generally, consequences due to LBP were more frequently reported than those due to NP or MBP. Regardless of area of complaint, care seeking and reduced physical activities were the most commonly reported consequences, followed by sick-leave, change of work, and disability pension. There was a small mid-life peak for care-seeking and a slow general increase in reduced activities with increasing age. Increasing age was not associated with a higher reporting of sick-leave but the duration of the sick-leave increased somewhat with age. Disability pension due to spinal pain was reported exceedingly rare before the age of 50. Typically, women slightly more often than men reported some kind of consequences due to spinal pain.

**Conclusions:**

Most people reporting spinal pain manage without any serious consequences. Low back pain more commonly results in some kind of consequence when compared to NP and MBP. Few age-related trends in consequences were seen with a slight predominance of women reporting consequences.

## Background

Low back pain (LBP) and neck pain (NP) are common in the general population [1-3] and are of considerable societal costs [4,5]. Whereas LBP and NP have been extensively studied, information on pain in the thoracic spine still remains relatively sparse [6].

Typically, the three spinal areas are studied or reported as separate entities, or as one single entity defined as just "back pain". Hence, only few studies have reported prevalence of pain in all three spinal regions simultaneously in the same population. This makes comparisons difficult as the designs and reporting of the studies differ considerably. Only a few population-based studies have included all three regions and simultaneously reported prevalence estimates for each of the spinal areas separately [7-13]. However, the presence of spinal pain provides only limited knowledge of how it affects the population at large. Of more relevance is what consequences spinal pain may have on people and to what extent they affect people's daily living.

Studies reporting consequences due to spinal pain for all three regions are also scarce. In adolescents, the most common consequences of spinal pain are reduced physical activity, seeking health care, and staying at home for a couple of days [10,12]. Similar findings have been found in adults [8]. Also in Danish elderlies aged 70-102 with NP or back pain, seeking treatment and reduced activities were relatively common [13].

Thus, it appears that the types of consequences due to spinal pain are fairly similar in different averaged age groups. However, it is not known if or how these consequences of spinal pain change with age as there may be age-related turning points. For example, in adults with LBP, a large Norwegian register-based study on the 1-yr incidence of sick-leave of at least 2 weeks' duration showed a slight but non-significant increase with age [14], whereas in another Norwegian population-based study, the 1-yr prevalence of long-term sick-leave due to LBP increased significantly from 5% in the 20-22 yr olds to 24% in the 60-62 yr olds [15].

Generally, women are overrepresented in the reporting of musculoskeletal pain [16] and potentially at greater risk of suffering from more consequences of pain compared to men. For example, in Norway and Sweden women were almost twice as likely as men to obtain a disability pension for musculoskeletal disorders [14,17]. In addition, a Danish study of older twin individuals showed that women with NP were more likely than men to reduce their physical activity and to seek health care [13].

It thus becomes apparent that the various consequences of pain in different spinal regions may have a significant socioeconomic burden to the society at large. However, there is still a need to further elaborate on the previous findings for all spinal regions, in particular for the thoracic region, as very little is known about the consequences of having a painful thoracic spine. It is also not known if pain in the different spinal regions result in different consequences, as only few studies [8,10] on consequences of all three spinal regions have been conducted. In addition, as some differences in consequences have been noted between adolescents and adults as well as between men and women it is relevant to investigate further how consequences are influenced by age and gender in all three spinal regions.

The purpose of this study was therefore to describe the prevalence of consequences in relation to age and gender for pain reported in the lumbar, thoracic and cervical regions in a large Danish population, representative of the general population.

## Methods

### Study design

This was a cross-sectional questionnaire-based omnibus study of a general population of twins from the Danish Twin Registry. Previous studies have shown that the Danish twin cohort is similar to the general population in many aspects [18]. Thus, population-based twin surveys from the Danish Twin Registry can be successfully used in epidemiologic studies. Details of this unique registry can be found in Skytthe et al's paper [19] and additional information can be obtained from the website http://www.sdu.dk/dtr.

In 2002 a postal survey entailing a 20 page questionnaire on a variety of different health related issues was sent out to all Danish twins aged 20 to 71 years who had previously accepted to participate. The data collection and the representativeness of the study have been extensively described elsewhere [7]. The study was approved by the Regional Scientific Ethics Committee and by the Data Protection Agency (file number: 20010201).

The questions on spinal pain were based on the Standardized Nordic Questionnaire on Musculoskeletal symptoms including mannequins with each of the three spinal regions clearly shaded [20]. However, in order to cut down on the number of pages in our questionnaire minor lay-out changes were made compared to the original questionnaire [7] and is available in Additional file 1. In the present study, the independent variables "pain ever" and "pain past year" were used in relation to pain in the lumbar, thoracic and cervical regions. The dependent dichotomous ('yes/no') variables for consequences of spinal pain in relation to "pain past year" were: 1) sick-leave (subsequently categorised as '1-7 days', '8-30 days' and ' > 30 days'), 2) reduced physical activity, and 3) care seeking. In relation to "pain ever", the following dichotomous data ('yes/no') were obtained: 4) changed work or tasks at work, and 5) under consideration for a disability pension or already on a disability pension. Individuals who reported none of the five consequences were classified as having "no consequences". The work-related variables (i.e. sick-leave and job changes) were only applied to work-active individuals.

### Data analysis and presentation

A systematic data cleaning resulted in less than 1% missing data for the individual pain and consequence-variables [7]. A detailed description of the age- and gender distribution of the various spinal pain patterns [7] as well as the effects of genetics on spinal pain in this cohort have previously been published [21].

The overall prevalence of each of the five consequences was calculated and presented as Table 1) for the whole study sample and 2) for those with spinal pain. For respondents with pain, the prevalence of each type of the five consequences was then calculated for each of the three spinal regions and graphically presented 1) by age and 2) by gender and age. As the study sample consisted of 34,902 individuals there was a probability that even small and biologically insignificant differences would obtain traditional statistical significance. Differences between groups were therefore determined solely by the presence of non-overlapping 95% confidence intervals. For the graphs, differences between these were considered significant if they were not overlapping over an interval of minimum 5 years. Hence, no additional statistical analyses were conducted. This paper consists of numerous estimates and so the results will mainly be explanatory and relating to general observations.

**Table 1:** Consequences of spinal pain

Type of consequence	Lumbar area	Thoracic area	Cervical area
Sought care past year	17	6	14
Reduced daily activities past year	17	4	9
Sick-leave past year	10	2	5
Number of days on sick-leave past year (1-7 d/8-30 d/> 30 d)	5/3/1*	1/1/0	3/2/1*
Changed work or work duties ever	8	2	4
Disability pension ever or under consideration for one	3	1	1

* The percentages have been rounded off and therefore do not add up to the total percentage of sick-leave.

The percentage of all study participants who reported some sort of consequence because of spinal pain in a study of 34,902 Danish twin individuals aged 20-71 (The 95% CI range for all consequences never exceeded 1 and is thus not included).

## Results

### Study sample

A comprehensive description of the study sample can be found in our previous paper [7]. In brief, a total of 34,902 valid questionnaires (74%) were returned after one reminder. There were slightly more women (54.4%) than men in the final study sample. The relative proportion of responders was fairly similar across all ages in the targeted study sample. The largest single age cohort (the 38-yr olds) consisted of 918 individuals and the smallest single age cohort (the 71-yr olds) of 164 individuals. Sixty-eight percent were work active and between the ages of 27 and 60 there were at least 400 work-active for each year of age (data not shown).

### The prevalence of self-reported spinal pain

A detailed analysis of self-reported spinal pain can be found in our previous paper [7]. In brief, 69% reported having had some sort of spinal pain during some time in their life and 55% in the past year. LBP in the past year was most frequent (43%) followed by NP (32%), whereas MBP was much less common (13%). Of those who reported spinal pain within the past year, 47% had pain in more than one spinal area.

### The prevalence of consequences in the whole study sample

Consequences due to spinal pain were most often seen for LBP followed by NP and MBP (Table 1). Health care seeking and reduced daily activities were the two most common consequences of spinal pain. Of all the participants, 17% had sought care for LBP in the past year, 14% for NP and 6% for MBP. Ten percent had been on sick-leave because of LBP, 5% because of NP and 2% because of MBP. Regardless of the site of pain, most people reported less than 8 days of sick-leave, whereas less than 1% had more than 30 days of sick-leave. The least common types of consequences were change of work/work duties and disability pension. Only 3% were either on or under consideration for disability pension because of LBP, and 1% because of NP or MBP, respectively.

### The prevalence of consequences among those with spinal pain

Overall, the majority (63%) of individuals with spinal pain did not report any consequence. Seventeen percent reported that spinal pain resulted only in a single consequence. Two, three, four, and five consequences were reported by 10%, 6%, 2.5%, and 0.5%, respectively. With few exceptions, consequences were generally more often reported by women and in individuals with LBP. Care seeking and reduced daily activities were the most commonly reported consequences, followed by sick-leave, change of work and disability pension (for further details see below and Table 2).

**Table 2:** Consequences of spinal pain by gender

Type of consequence in relation to area of pain	All	Men	Women
*Sought care last year because of ...*			
LBP	38 (37-39)	36 (35-37)	39 (38-40)
MBP	39 (38-41)	34 (32-36)	42 (40-44)
NP	40 (39-41)	34 (32-35)	43 (42-44)
*Reduced physical activity last year because of...*			
LBP	39 (38-39)	40 (38-41)	38 (37-39)
MBP	29 (27-30)	28 (26-31)	29 (27-31)
NP	28 (27-29)	25 (24-27)	29 (28-30)
*Sick-leave last year because of...*			
LBP	23 (22-24)	24 (23-26)	21 (20-22)
MBP	17 (15-18)	18 (16-20)	16 (14-17)
NP	15 (15-16)	14 (13-15)	16 (15-17)
*Ever changed work/work duties because of...*			
LBP	14 (13-14)	13 (12-13)	14 (14-15)
MBP	11 (10-12)	9 (8-10)	13 (12-14)
NP	10 (9-10)	7 (6-8)	11 (10-12)
*Has a disability pension or under consideration for one because of...*			
LBP	4 (4-5)	3 (3-4)	5 (5-6)
MBP	5 (4-5)	3 (2-4)	6 (5-6)
NP	4 (3-4)	2 (2-3)	4 (4-5)

LBP = low back pain; MBP = mid back pain; NP = neck pain.

The percentage of study participants with spinal pain in the past year who reported some sort of consequence in a study of 34,902 Danish twin individuals aged 20-71 (95% confidence intervals).

#### Sought care

**Figure 1:**
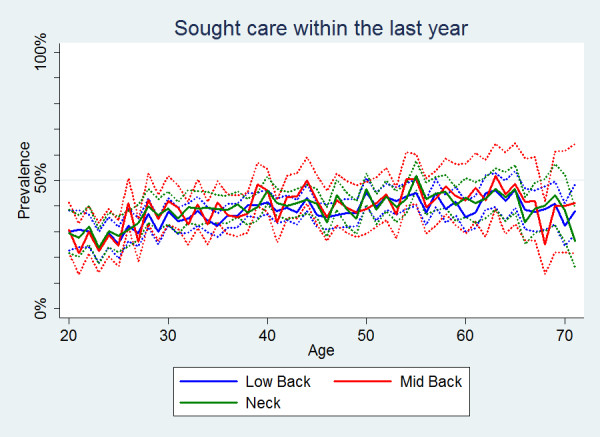
**Sought care within the last year**. The proportions of individuals with spinal pain in the past year who sought care in the past year by age according to data from a Danish omnibus survey (n = LBP: 15,093; MBP: 4,535; NP: 11,316).

**Figure 2:**
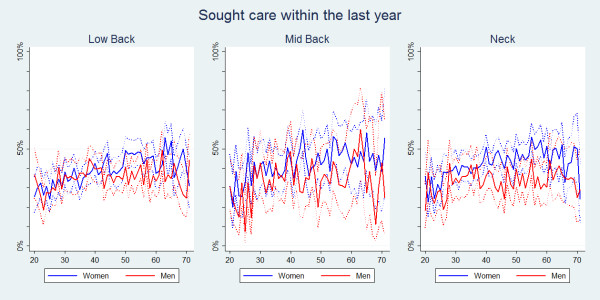
**Sought care within the last year**. The proportions of individuals with spinal pain in the past year who sought care in the past year by age and reported separately for men and women according to data from a Danish omnibus survey (n = LBP: 15,093; MBP: 4,535; NP: 11,316).

Regardless of the area of pain the proportions of individuals seeking care were the same and with a slight peak in the middle years (Table 2, Figure 1). Common for all three spinal areas was that women were generally more likely than men to seek care, although the confidence intervals were overlapping (Figure 2).

#### Reduced physical activities

**Figure 3:**
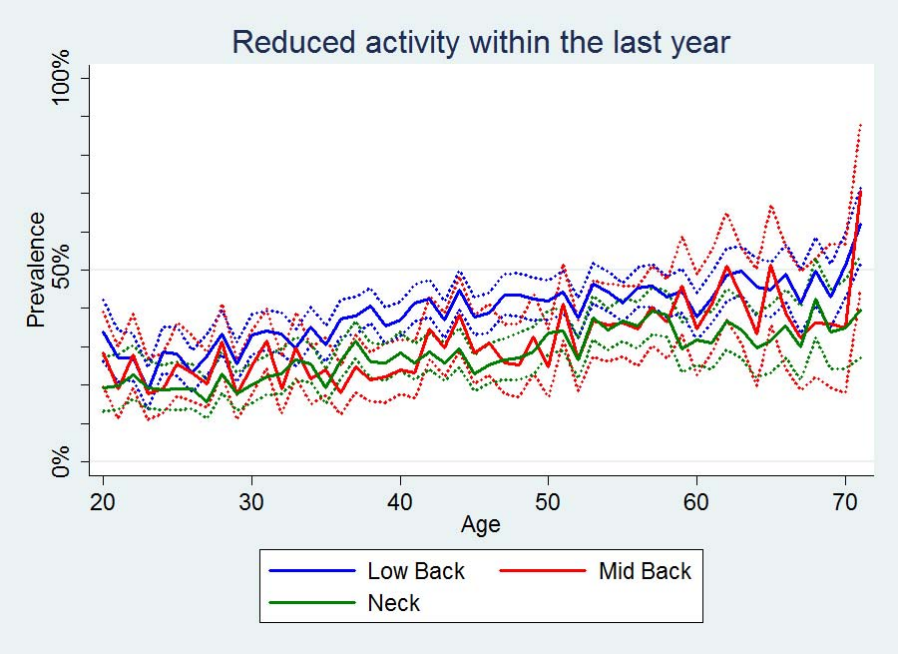
**Reduced activity within the last year**. The proportions of individuals with spinal pain in the past year who reduced their physical activity in the past year by age according to data from a Danish omnibus survey (n = LBP: 15,093; MBP: 4,535; NP: 11,316).

**Figure 4:**
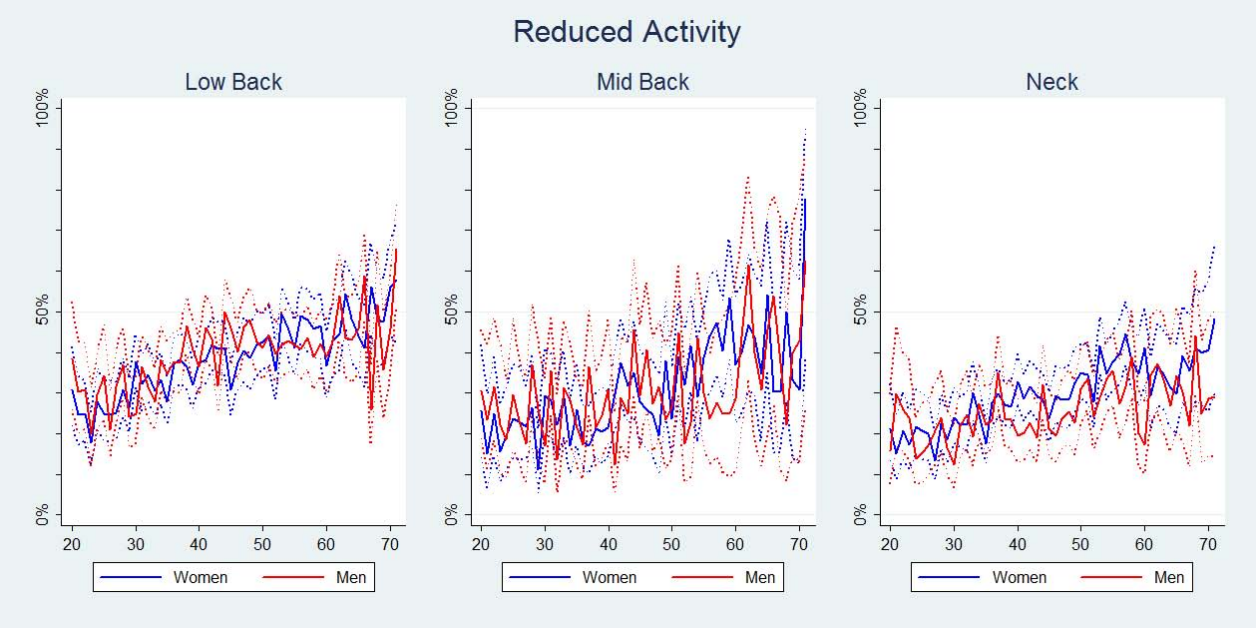
**Reduced activity within the last year**. The proportions of individuals with spinal pain in the past year who reduced their physical activity in the past year by age and reported separately for men and women according to data from a Danish omnibus survey (n = LBP:15,093; MBP:4,535; NP:11,316).

Reduced physical activities were significantly more common in people with LBP than in people with MBP or NP (Table 2). For all three pain areas, the proportion of reduced physical activities increased slightly with age (Figure 3). There were no gender differences for LBP and MBP (Figure 4), whereas for NP, overall women reduced their physical activities more often than men even though, again, this was not statistically significant (Table 2).

#### Sick-leave

**Figure 5:**
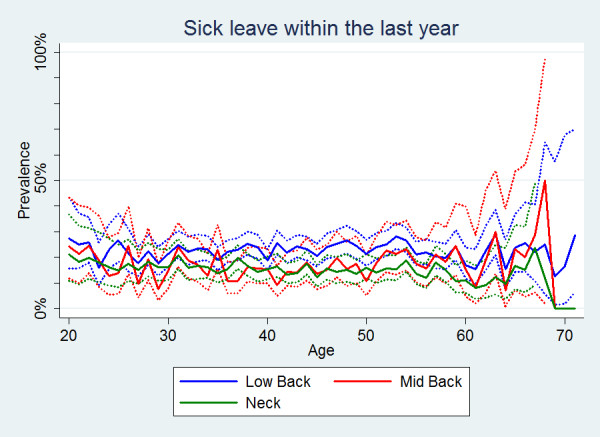
**Sick leave within the last year**. The proportions of individuals with spinal pain in the past year who took some sick-leave in the past year by age according to data from a Danish omnibus survey (n = LBP: 10,578; MBP: 3,028; NP: 7,958).

**Figure 6:**
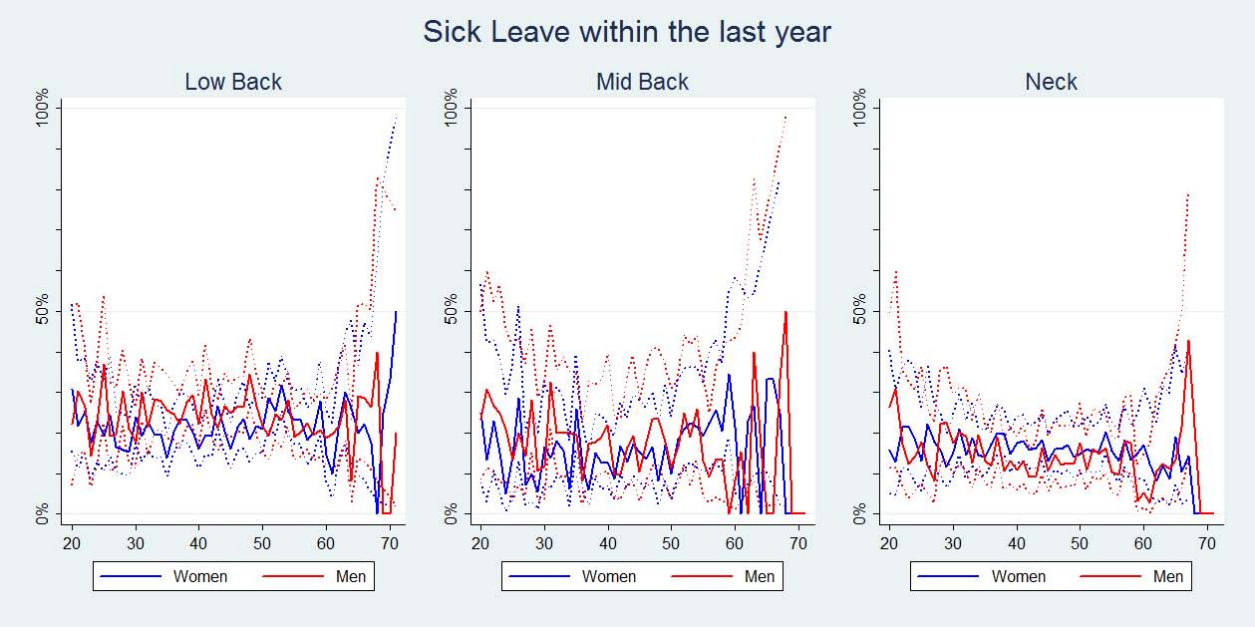
**Sick leave within the last year**. The proportions of individuals with spinal pain in the past year who took some sick-leave in the past year by age and reported separately for men and women according to data from a Danish omnibus survey (n = LBP:10,578; MBP: 3,028; NP:7,958).

Sick-leave was also significantly more common in people with LBP (Table 2). The prevalence estimates for each of the three pain sites were fairly similar across all ages (Figure 5). Overall, men significantly more often reported sick-leave than women (Table 2), especially for the young and middle aged men (Figure 6). For NP, women had the highest estimates predominantly from the age of 35 and onwards (Figure 6). No clear age trend was noted for MBP.

#### Distribution of duration of sick days

**Figure 7:**
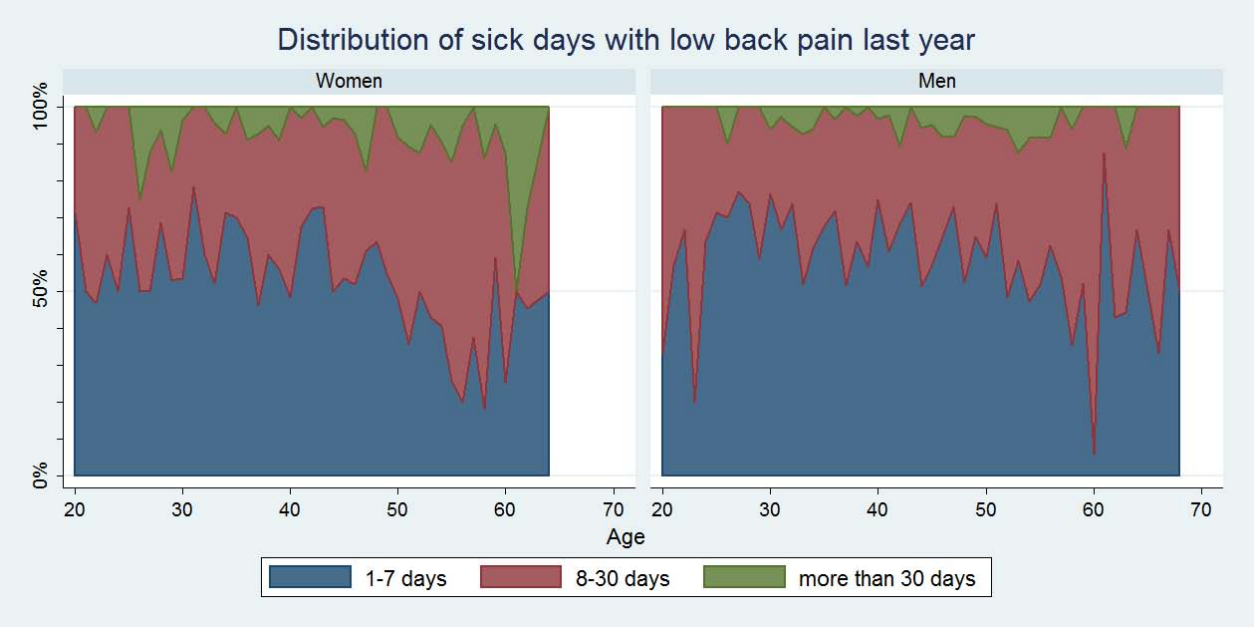
**Distribution of sick days with low back pain within the last year**. Categorised distributions of sick-leave durations among those who took sick-leave in the past year by age and reported separately for men and women according to data from a Danish omnibus survey (n = 2,409).

**Figure 8:**
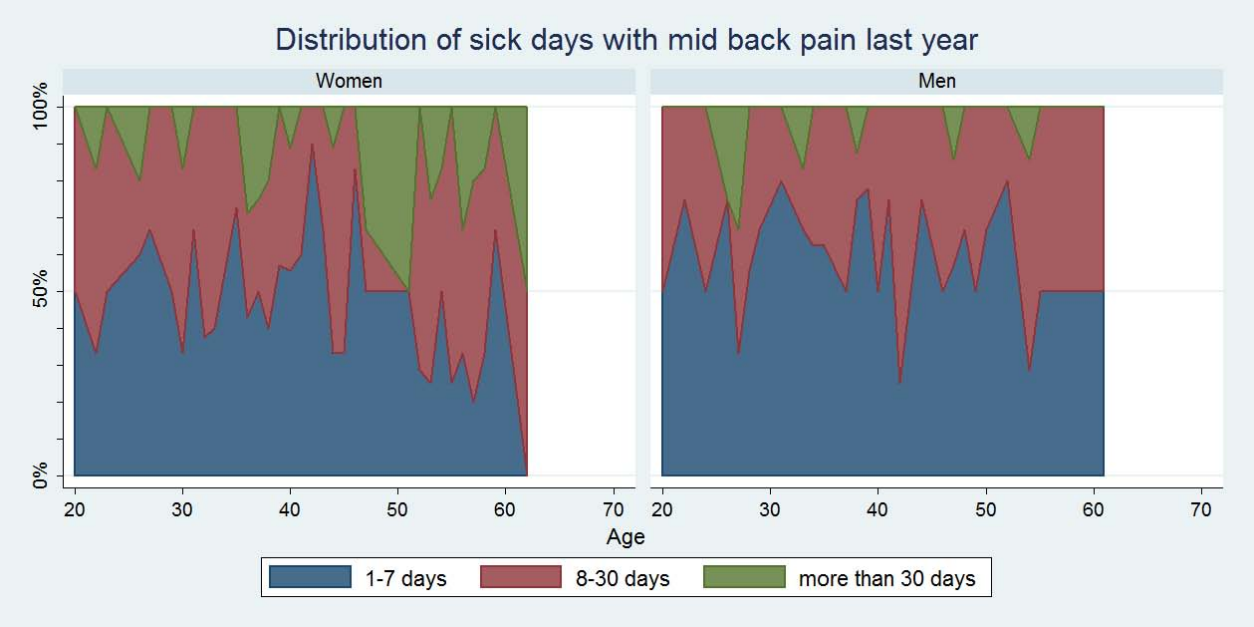
**Distribution of sick days with mid back pain within the last year**. Categorised distributions of sick-leave durations among those who took sick-leave in the past year by age and reported separately for men and women according to data from a Danish omnibus survey (n = 2,409).

**Figure 9:**
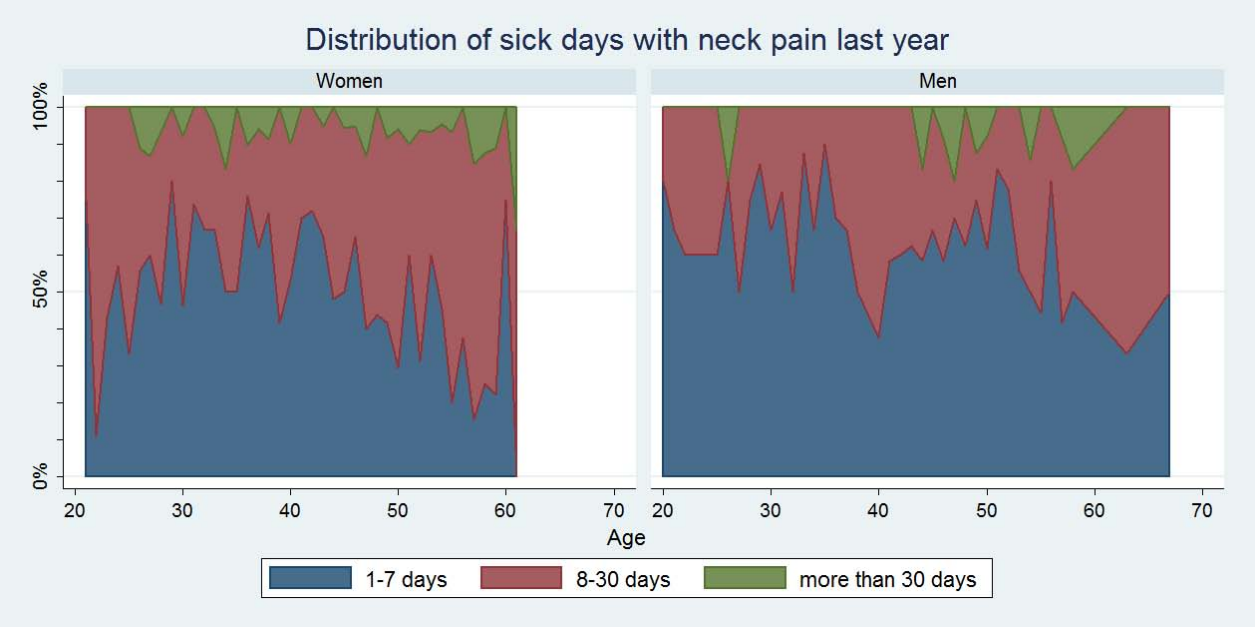
**Distribution of sick days with neck pain within the last year**. Categorised distributions of sick-leave durations among those who took sick-leave in the past year by age and reported separately for men and women according to data from a Danish omnibus survey (n = 2,409).

Some variations for the distribution of sick days were seen across age groups and for each spine site (Figure 7, 8, 9). Regardless of the area of pain, 1-7 days was generally the most common sick-leave period up till the age of approximately 50. From that age, a sick-leave period of 8-30 days became more common, whereas a sick-leave of >30 days remained relatively uncommon throughout all ages. In general, women were somewhat more likely to report longer sick-leave than men.

#### Changed work or duties at work

**Figure 10:**
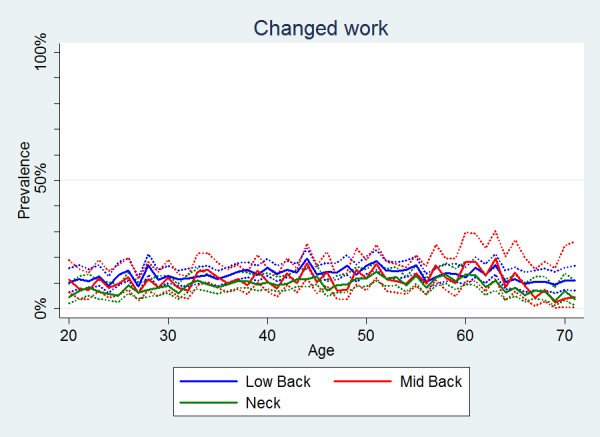
**Changed work within the last year**. The proportions of individuals with spinal pain ever who ever changed their work or work duties by age according to data from a Danish omnibus survey (n = BP: 20,053; MBP: 5,966; NP: 14,059).

**Figure 11:**
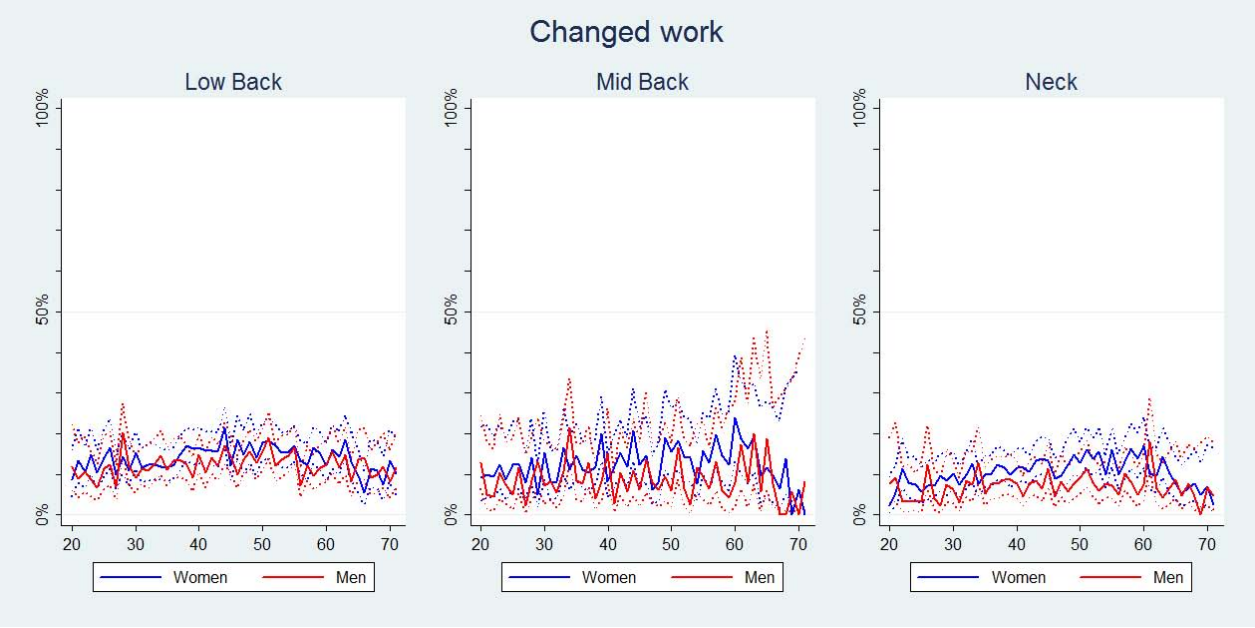
**Changed work within the last year**. The proportions of individuals with spinal pain ever who changed their work or work duties by age and reported separately for men and women according to data from a Danish omnibus survey (n = LBP: 20,053; MBP: 5,966; NP: 14,059).

Individuals with LBP were more likely to change work or work duties when compared to individuals with MBP or NP (Table 2). The prevalence estimates for each of the three spinal regions were similar across all ages (Figure 10). Overall, significantly more women than men changed their work because of spinal pain (Table 2, Figure 11).

#### Disability pension

**Figure 12:**
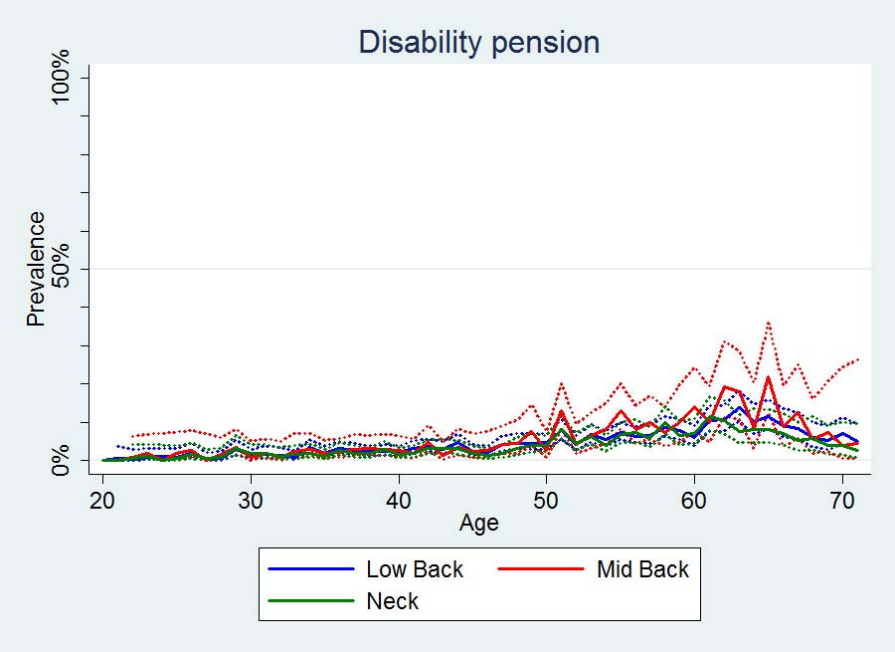
**Seeking or being on disability**. The proportions of individuals with spinal pain ever who had a disability pension or were under consideration for one by age according to data from a Danish omnibus survey (n = LBP:20,053;MBP:5,966;NP:14,059).

**Figure 13:**
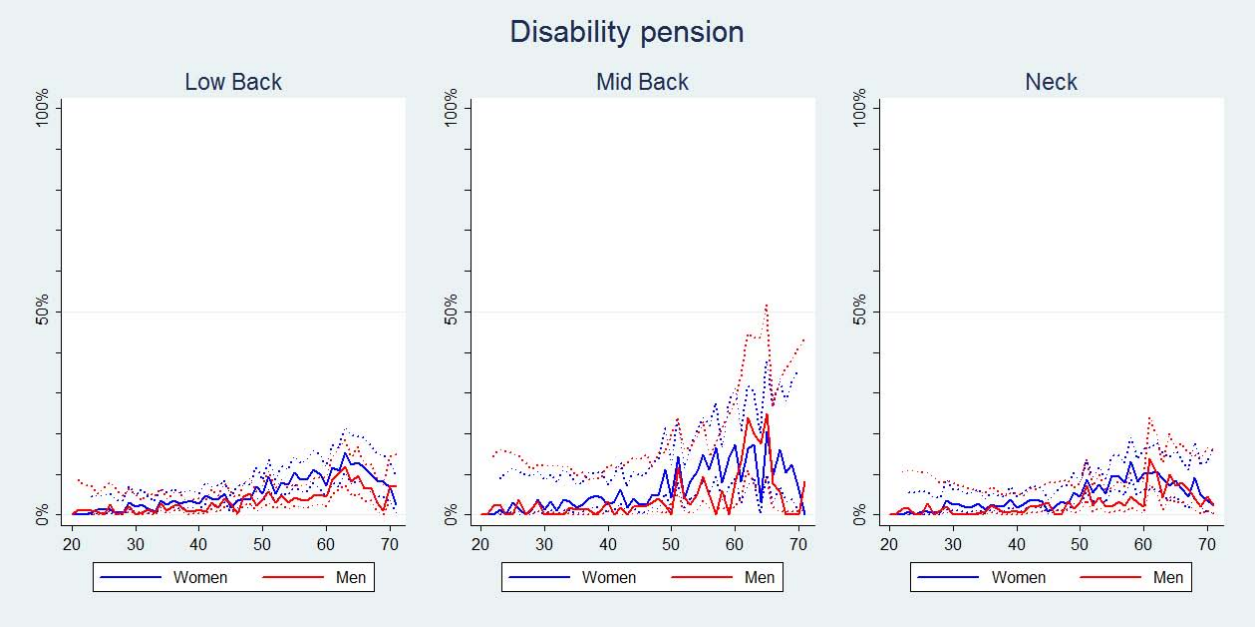
**Seeking or being on disability**. The proportions of individuals with spinal pain ever who had a disability pension or were under consideration for one by age and reported separately for men and women according to data from a Danish omnibus survey (n = LBP:20,053;MBP:5,966;NP:14,059).

Although there were no significant differences between the estimates on disability pension for the areas of pain (Table 2), the estimates for MBP were consistently higher (Figure 12). Seeking disability pension because of spinal pain remained uncommon until the age of approximately 50 where there was a marked increase. Overall, disability pension was significantly more often reported by women than by men (Table 2, Figure 13).

### In summary

#### In general

• Almost two-thirds of individuals with spinal pain did not report any consequence.

• Care seeking and reduced physical activities were the most commonly reported consequences, followed by sick-leave, change of work and disability pension.

• Consequences due to LBP were more often reported than consequences due to MBP and NP.

#### In relation to age

• Overall, no dramatic age effect was found for any of the spine areas in relation to any of the consequences.

• There was a small mid-life peak for care-seeking and a slow general increase in reduced activities with increasing age, whereas sick-leave and change of work did not show any age-related differences.

• Typically the duration of sick-leave was 1-7 days, but from the age of 50 the sick-leave period of 8-30 days became more prevalent.

• Sick-leave of >30 days remained relatively uncommon throughout all ages.

• Disability pension was extremely uncommon before the age of 50.

#### In relation to gender

• Generally, women somewhat more often than men reported some kind of consequence as well as longer periods of sick-leave.

## Discussion

### General findings

As expected, spinal pain does not lead to any consequences for most people. Thus, most people with spinal pain seem to manage quite well, either because the pain intensity is only mild or because they have sufficient coping strategies. Still, even if most people do not report more common consequences such as sick-leave because of pain it is not known how they are affected on a daily basis. For instance, persons with back pain are known to turn up at work where they may not perform as well as what they would normally do [22].

Given that almost two-thirds of people with spinal pain do not report any of the five consequences it may suggest that spinal pain is not an important public health problem. However, even if only a third of spinal pain sufferers report some kind of consequence it would still have a significant impact on the health care system as more than half of the study sample reported some kind of spinal pain within the past year. In addition to this, any sick-leave, even if only for shorter durations, along with reduced daily activities, may result in substantial indirect costs to society.

Among those who did take action, the most common consequences were care-seeking and reduced daily activities. In Danish school-children, seeking health care was also more common than reduction in physical activities [10]. In Danish elderly individuals aged 70 or more, care-seeking is also more common than reduction of physical activities [13]. This may indicate that there is a subgroup of people who rely more on external assistance than on their own strategies and hence take action when they have spinal pain.

The health care system in Denmark is either available free of charge or is to a large extent reimbursed. Therefore, it would be tempting to think that the hierarchy of consequences found in this study is typical of what people with back pain would do if medical treatment is free of charge and also, as in Denmark, if unemployment is not a major issue. However, a similar hierarchy of consequences was found also in a Swedish study [23], despite differences between the countries both on the fee structure and unemployment rates. We are unaware of any other similar hierarchical studies conducted in non-Nordic countries thus, making it difficult to compare these hierarchal trends to socio-economic factors.

Differences were noted between the three spinal regions. Not surprisingly, consequences due to LBP were generally more commonly reported than for any other spinal pain site mainly because more individuals suffered from LBP. However, even when the relative frequencies were calculated, reduced physical activity, sick-leave and change of work remained significantly higher for individuals with LBP compared to MBP and NP. Thus, it seems that LBP has a greater impact on daily living and that individuals with LBP have slightly different coping strategies compared to those with MBP or NP.

### In relation to age

We have previously shown that spinal pain is fairly evenly spread over all ages, with no obvious increase in prevalence towards the elderlies [7]. Still, given the general age-related degenerative spinal changes an accumulation of various consequences in the older age groups would be expected. One could therefore assume that the more serious consequences, i.e. longer sick-leave, change of work, and disability pension would occur mainly in older people and that these would be preceded by reduced physical activities, care-seeking and shorter sick-leave in the younger age groups. However, this was only partly demonstrated in our study.

Interestingly, the number of individuals on sick-leave does not increase with increasing age, but rather the duration of sick-leave increases. Thus, it may be that those individuals who suffer from spinal pain at an earlier age will get worse over time in terms of pain intensity and/or because their general capability to cope with spinal pain decreases with age.

Furthermore, in our study no age-related trend in change of work due to spinal pain was noted. This was unexpected as one would normally assume a certain degree of accumulation with age as people "wear down" because of spinal pain. A theory could be that people with spinal pain at an early age may already realise that they need to change work or work duties because of pain. As expected, disability pension was extremely uncommon in the younger people, but it did not increase dramatically in the oldest group.

Admittedly, memory decay could result in artificially low estimates when the denominator related to spinal pain ever (change of work and disability pension). It is therefore possible that the change of work estimate in reality should be higher, whereas this is unlikely for disability.

### In relation to gender

Not surprisingly, more women generally report the presence of spinal pain as well as consequences due to spinal pain compared to men. This sex or gender difference in musculoskeletal pain is well documented [16,24] and is probably due to biological influences (i.e. hormonal), psychosocial factors (i.e. gender role expectations) or a combination of both factors (e.g. coping/catastrophizing/anxiety) [24]. It is beyond the scope of this paper to further discuss this separate research area, but the readers are encouraged to read the comprehensive review by Fillingim et al [24].

### Limitations

The strengths and weaknesses of this study have been discussed before [7], to which should be added that misclassification of our consequence variables is possible, and that this cannot be verified. Under reporting would probably be more likely than over reporting as the 30-page questionnaire is not specifically focused on spinal pain. We have therefore no reasons to believe that we have a specially selected group of individuals with spinal pain in our study. If, however, this is the case the effect the results will probably be biased towards the null, although this cannot be determined.

### Future perspectives

While not studied here, other aspects of interest such as similarities and differences between the spinal regions, multiple pain sites, specific spinal conditions, co-morbidities, various psychosocial factors and their interactions need to be further investigated in order to understand the true underlying nature of the consequences of spinal pain.

## Conclusions

Most people with spinal pain manage without any serious consequences. LBP more commonly results in some kind of consequence compared to NP and MBP. Few age-related trends in consequences were seen with a slight predominance of women reporting consequences. Increasing age is not associated with a higher reporting of sick-leave but rather the duration of the sick-leave increases somewhat with age

## Competing interests

The authors declare that they have no competing interests.

## Authors' contributions

All authors read and approved the final manuscript. KOK was responsible for the epidemiologic study. JH, RF and CLY secured funding for the back pain study. CLY and JH formulated the preliminary research questions and designed the spinal pain questionnaire. CLY formulated the research questions for the present analyses. JN analyzed the data and provided the graphical presentations. CLY and RF did the data interpretation. CLY and RF wrote the first draft and all contributed to the final version.

## Pre-publication history

The pre-publication history for this paper can be accessed here:

http://www.biomedcentral.com/1471-2474/12/39/prepub

## Supplementary Material

Additional file 1 **English translation of the questions on back pain used in the Danish omnibus study**. A two-page Word 2003 document with an English translation of the questions on back pain used in the Danish omnibus study. Click here for file
