# Supplementary material for: Consequences of spinal pain: Do age and gender matter? A Danish cross-sectional population-based study of 34,902 individuals 20-71 years of age
Source: BMC Musculoskelet Disord. 2011 Feb 8;12:39. doi: 10.1186/1471-2474-12-39 (PMC3041727; doi:10.1186/1471-2474-12-39)
Supplement: Additional file 1 — English translation of the questions on back pain used in the Danish omnibus study. A two-page Word 2003 document with an English translation of the questions on back pain used in the Danish omnibus study. [file 1471-2474-12-39-S1.DOC]

### Additional File 1. English translation of the questions on back pain used in the Danish omnibus study. Original version available from the authors on request.

Below follow some questions on trouble in the neck, mid back and low back. With trouble is meant pain and other discomfort in the low back, mid back and or neck (see drawings).

[Here are inserted 3 mannekins from the Nordic questionnaire, each showing one of the three spinal regions, which in total covered the whole spine]

Have you ever had:

Low back pain yes/no

Mid back pain yes/no

Neck pain yes/no

For how long have you during the last 12 months altogether had:

(write 0, if you did not have any problems within the last 12 months)

Low back pain approx. ….. days

Mid back pain approx. ….. days

Neck pain approx. ….. days

Have you during the last 12 months had pain radiating from:

Low back into leg/legs yes/no

Mid back into chest yes/no

Neck pain into arm/arms yes/no

Have you during the last 12 months reduced your physical activity at work or during leisure time because of:

Low back pain yes/no

Mid back pain yes/no

Neck pain yes/no

Have you ever changed work or tasks at work because of:

Low back pain yes/no

Mid back pain yes/no

Neck pain yes/no

For how long have you during the last 12 months altogether not been able to perform your work because of:

Low back pain approx. ….. days

Mid back pain approx. ….. days

Neck pain approx. … .. days

Have you during the past 12 months been examined or treated by a medical doctor, chiropractor, physiotherapist or other because of:

Low back pain yes/no

Mid back pain yes/no

Neck pain yes/no

Are you presently under consideration for or do you have a disability pension because of:

Low back pain yes/no

Mid back pain yes/no

Neck pain yes/no
